# “Would you like a flu shot with your order?”—A coronavirus disease 2019 (COVID-19) pandemic drive-through response to address delayed pediatric immunization in Detroit, Michigan

**DOI:** 10.1017/ice.2020.1410

**Published:** 2021-01-05

**Authors:** Eric J. McGrath, Donia Dalal, Lynn Smitherman, Sharon Marshall, Christopher Youngman, Charles J. Barone, Herman Gray, Najibah Rehman, Elizabeth Secord

**Affiliations:** 1 Department of Pediatrics, Wayne State University School of Medicine, Detroit, Michigan; 2 Detroit Health Department, Detroit, Michigan; 3 Department of Pediatrics, Henry Ford Hospital, Detroit, Michigan

*To the Editor—*Since the coronavirus disease 2019 (COVID-19) pandemic and associated “shelter in place” orders in the spring of 2020, numerous children in the United States,^1^ but especially in Michigan,^2^ have not received the recommended immunizations. Furthermore, baseline Michigan vaccination rates have been low. Here, we describe an effort to address this public health crisis within the COVID-19 crisis.

A “drive-through” immunization fair^3–6^ was held Saturday, October 10, 2020, in which parents and their children (aged 6 weeks to 18 years) stayed in their vehicles and all participants >2 years old wore required facemasks. The Wayne Pediatrics (WP) clinical group, affiliated with Wayne State University (WSU) School of Medicine (SOM), collaborated with the Detroit Health Department (DHD) to offer the event. Parents were encouraged to call the DHD to schedule a drive-through appointment before the day of the event, but patients who showed up to the event without an appointment were also seen. Before the event, local families were informed about the event through advertising with bulk mailings of postcards, by social media, by e-mail alerts to community partners, and by other widespread marketing publicity.

**Fig. 1.**
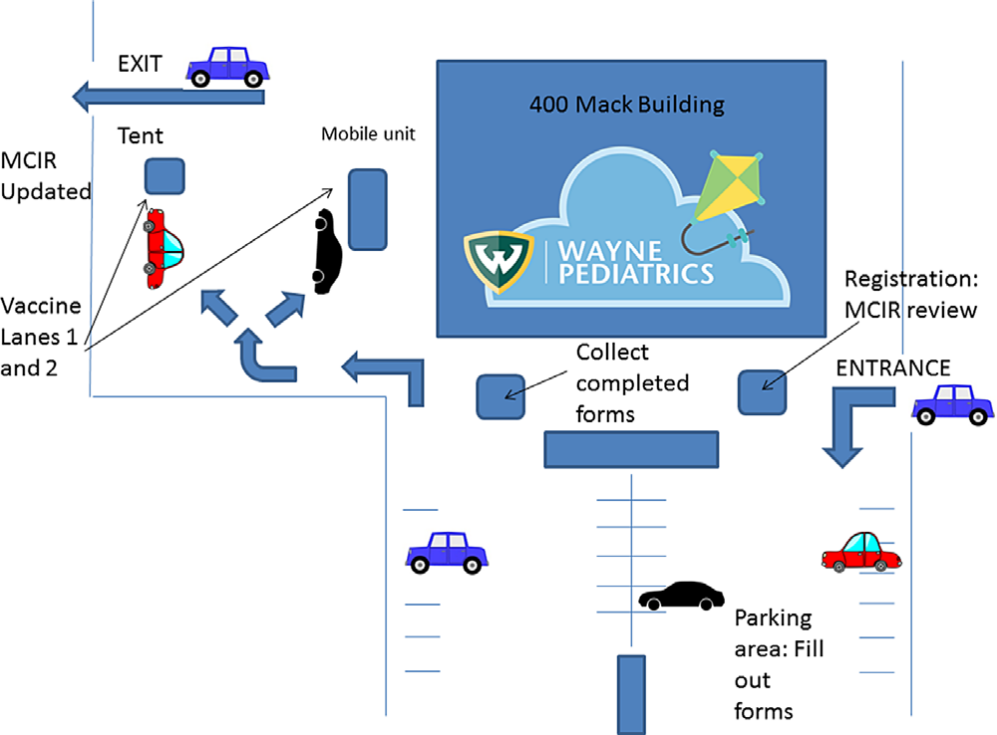
The COVID-19 pandemic drive-through immunization event traffic flow diagram.

Routine vaccines from the 2020 pediatric schedule^7^ were offered to participants due for immunization or requesting influenza vaccine. Immunizations were supplied by the DHD. The WP clinic-building parking lot was used for one-way traffic flow (Fig. 1). Henry Ford Pediatrics donated their pediatric mobile vehicle and professional driver for the event. Older children could receive vaccines in their deltoid or shoulder though their vehicle window, and the mobile unit was used for privacy for infants and very young children vaccinates in the upper thigh.

The DHD dedicated staff members included an immunization coordinator, 2 nurses, 2 patient navigators, and 2 registration staff. The WP clinic staff volunteers included a nurse, 4 medical assistants, the clinical manager, a clinical supervisor, 2 medical secretaries, and 6 physicians. Each completed a 4.5-hour shift for the 12-hour event. Furthermore, 19 WSU SOM second-year medical students trained in PPE donning and doffing and immunization administration practices by in-person demonstration 2 days prior to the event; each completed a 2-hour volunteer shift during the event. A special events manager from WSU was present the entire day, and 3 WSU police officers helped with vehicle traffic.

At registration, the parent received a pen (to keep), intake paperwork, and a plastic (sanitizable) clipboard to complete required documentation. Parent identification and insurance cards were verified by DHD staff. The Michigan Care Improvement Registry (MCIR) vaccine record database was accessed for each participant with on-site, mobile computers and printers. The DHD staff reviewed the MCIR record to assess required or past-due immunizations. All participants remained in their vehicle while volunteer clinical staff and medical students picked up the completed forms. Finally, families were guided toward 1 of 2 drive-through lanes and were vaccinated by DHD nurses. The MCIR was updated in real-time, was printed, and was then given to the parent.

All volunteer medical and clinical staff with direct patient contact donned the following PPE: mask or respirator, face shield or goggles, gown (when vaccinating a participant), and gloves. Volunteers clearing an initial temperature check and paper-based COVID-19 symptom evaluation form then received ongoing temperature checks every 2 hours. Hand sanitizer was readily available for hand hygiene. All volunteer staff used clinic restrooms that were professionally cleaned after the event.

This event took place during the COVID-19 pandemic, with an emphasis on enhanced infection prevention methods for families and volunteers. During the event, 40 participants were successfully immunized.
